# The *In Vivo* Therapeutic Efficacy of the Oncolytic Adenovirus Delta24-RGD Is Mediated by Tumor-Specific Immunity

**DOI:** 10.1371/journal.pone.0097495

**Published:** 2014-05-27

**Authors:** Anne Kleijn, Jenneke Kloezeman, Elike Treffers-Westerlaken, Giulia Fulci, Sieger Leenstra, Clemens Dirven, Reno Debets, Martine Lamfers

**Affiliations:** 1 Dept of Neurosurgery, Brain Tumor Center, ErasmusMC, Rotterdam, The Netherlands; 2 Laboratory of Experimental Tumor Immunology, Dept of Medical Oncology, ErasmusMC, Rotterdam, The Netherlands; 3 Brain Tumor Research Center, Molecular Neurosurgery Laboratory, Department of Neurosurgery, Massachusetts General Hospital, Boston, Massachusetts, United States of America; University of Michigan School of Medicine, United States of America

## Abstract

The oncolytic adenovirus Delta24-RGD represents a new promising therapeutic agent for patients with a malignant glioma and is currently under investigation in clinical phase I/II trials. Earlier preclinical studies showed that Delta24-RGD is able to effectively lyse tumor cells, yielding promising results in various immune-deficient glioma models. However, the role of the immune response in oncolytic adenovirus therapy for glioma has never been explored. To this end, we assessed Delta24-RGD treatment in an immune-competent orthotopic mouse model for glioma and evaluated immune responses against tumor and virus. Delta24-RGD treatment led to long-term survival in 50% of mice and this effect was completely lost upon administration of the immunosuppressive agent dexamethasone. Delta24-RGD enhanced intra-tumoral infiltration of F4/80+ macrophages, CD4+ and CD8+ T-cells, and increased the local production of pro-inflammatory cytokines and chemokines. In treated mice, T cell responses were directed to the virus as well as to the tumor cells, which was reflected in the presence of protective immunological memory in mice that underwent tumor rechallenge. Together, these data provide evidence that the immune system plays a vital role in the therapeutic efficacy of oncolytic adenovirus therapy of glioma, and may provide angles to future improvements on Delta24-RGD therapy.

## Introduction

Patients harboring a malignant glioma have a dismal prognosis. Despite current therapy consisting of surgery, chemotherapy and radiotherapy, the median survival of glioblastoma (GBM, grade IV glioma) patients is 14.6 months [1]. New treatment modalities are necessary to improve this prognosis. Oncolytic viruses (OV), capable of replicating specifically in cancer cells, have shown promising results in various cancer models [2]–[5] and the first clinical trials have shown safety and feasibility of this treatment option [6]–[10]. The oncolytic adenovirus Delta24-RGD has shown potent antitumor activity in various preclinical studies [11]–[14] and is currently under investigation in phase I/II trials for recurrent glioblastomas [15], [16]. The backbone of this human serotype 5 adenovirus has a 24 base-pair deletion in the E1A gene, abrogating E1A binding to the retinoblastoma protein (RB) and rendering the virus tumor-specific. Additionally, an RGD peptide inserted into the fiber-knob allows the virus to anchor directly to αVβ3 and αVβ5 integrins, therewith improving infectability of integrin-positive GBM cells [14], [17].

Recent literature points toward the importance of the immune response during oncolytic therapy and evidence is accumulating that the immune response is essential for achieving therapeutic effects with virotherapy [18]–[24]. In fact, OVs have been reported to aid the immune system in mounting an anti-tumor response [19], [20], [22], [24], [25]. Conversely, it has been shown that the immune response hampers the efficacy of viral therapy by attacking the virus, and that by suppressing the immune system, viral replication and anti-tumor efficacy is enhanced [18], [21], [26]. Due to the presumed species-specificity of human adenoviruses, the efficacy of Delta24-RGD has in the past been assessed exclusively in human xenografts in immuno-compromised animal models [5], [27]–[29]. However, the use of these models precludes studies into the role of the immune system in adenoviral oncolytic virotherapy.

To address the above aspects for the Delta24-RGD virus in glioma, we implemented the immune competent syngeneic GL261 glioma model to investigate the role of the immune system during Delta24-RGD treatment of intracranial glioma. We show that Delta24-RGD can replicate and induce cytotoxicity in the murine glioma cell line GL261 [30] and therefore can be used as a model to study the contribution of the immune system in oncolytic adenoviral therapy of glioma. Our results demonstrate for the first time that Delta24-RGD treatment induces anti-viral and anti-tumor immune responses, resulting in long-term survival and lifetime protection against tumor rechallenge.

## Results

### Delta24-RGD Induces Cytotoxicity *In vitro* and Neutralizing Antibodies *In vivo*

**Figure 1:**
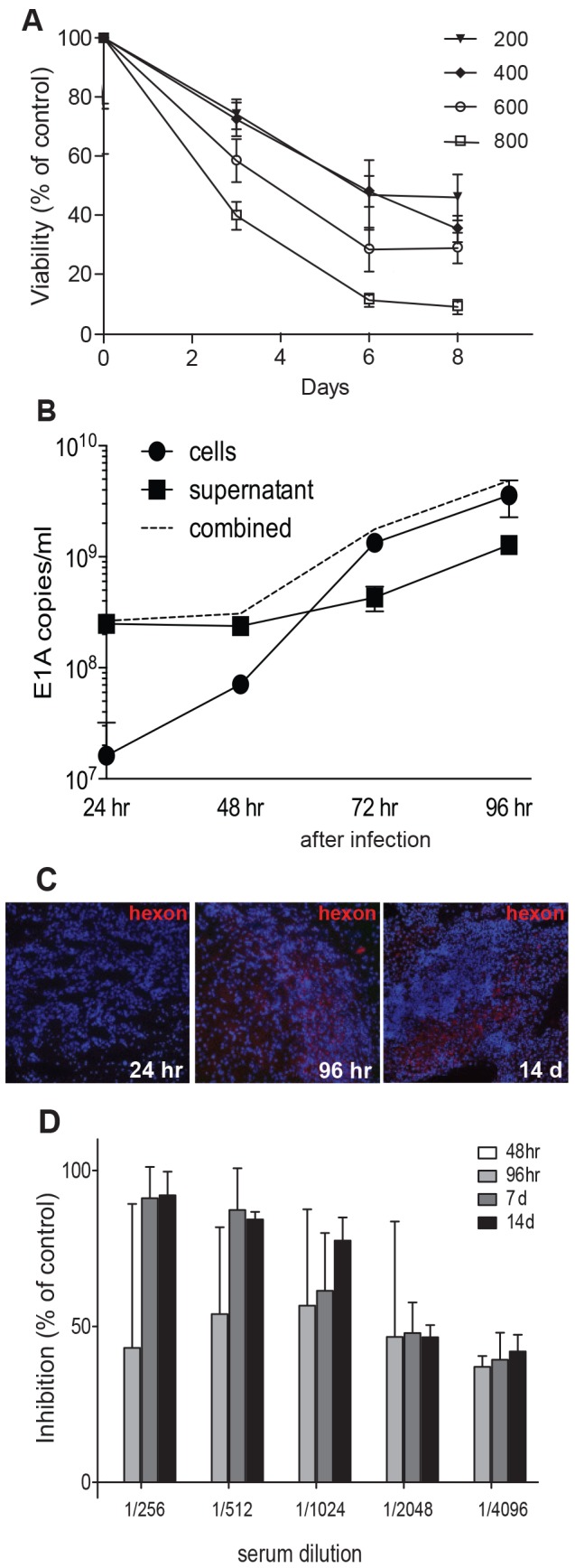
Delta24-RGD induces cytotoxicity *in vitro* and neutralizing antibodies *in vivo*. A) GL261 cells were seeded and infected in triplicate with a viral dose ranging from 200 to 800 MOI. Viability was measured using a WST-1 assay at day 3, 6 and 8 post viral infection. Results are expressed as mean percentage of non-treated controls. Error bars indicate SD. B) Quantitative PCR for E1A to assess the viral load in GL261 cells. Harvested cells and supernatant were measured after 24, 48, 72 and 96 hr. C) Immunohistochemical analysis of the adenoviral protein hexon in intracranial GL261 tumors harvested 24, 96 hr and 14 days post virus injection. Representative images of two mice per time-point are shown. D) Ad-luc-RGD was incubated on A549 cells in the presence or absence of serial dilutions of mouse sera derived from Delta24-RGD treated mice at indicated time-points. Results are presented as percentage of control ± SD.

To evaluate the susceptibility of murine GL261 cells to Delta24-RGD infection, GL261 cells were infected with a virus concentration range of 200–800 Multiplicity of Infection (MOI). This resulted in a dose-dependent decrease in cell viability, with approximately 50% cell death with MOI 200 Delta24-RGD at day 6 (figure 1A). As an indicator of viral replication of Delta24-RGD in GL261 cells *in vitro*, viral E1A levels were determined by qPCR between 24–96 hr post-infection at MOI 100, showing an approximate 100-fold increase in E1A copies in the cells and a 10-fold increase in the supernatants between 48 and 96 hr (figure 1B). *In vivo* replication was assessed by injection of Delta24-RGD into intracranial GL261 tumors. Analysis of tumors revealed the presence of adenoviral hexon at 96 hr and 14 days post injection, suggesting ongoing viral activity for at least 2 weeks (figure 1C).

One of the hallmarks of anti-viral immunity is the production of neutralizing antibodies against the virus. To evaluate this in our model, sera were sampled from mice that received intratumoral Delta24-RGD-injection. The infection efficiency of the luciferase-encoding adenoviral vector Ad-luc-RGD was assessed on A549 cells in the presence or absence of mouse serum obtained at 48, 96 hours, 7 or 14 days post treatment (figure 1D). At 48 hours, no inhibition of viral infection was observed. Starting at 96 hours, neutralizing antibodies in the sera of virus-treated mice inhibited the infection, albeit at variable levels between the mice At 7 and 14 days, high levels of neutralizing antibodies were present in all mice, completely blocking adenoviral infection (figure 1D). Sera derived from PBS-treated mice did not inhibit Ad-luc-RGD infection (results not shown).

### Immunosuppressive Treatment Inhibits *In vivo* Efficacy of Delta24-RGD

**Figure 2:**
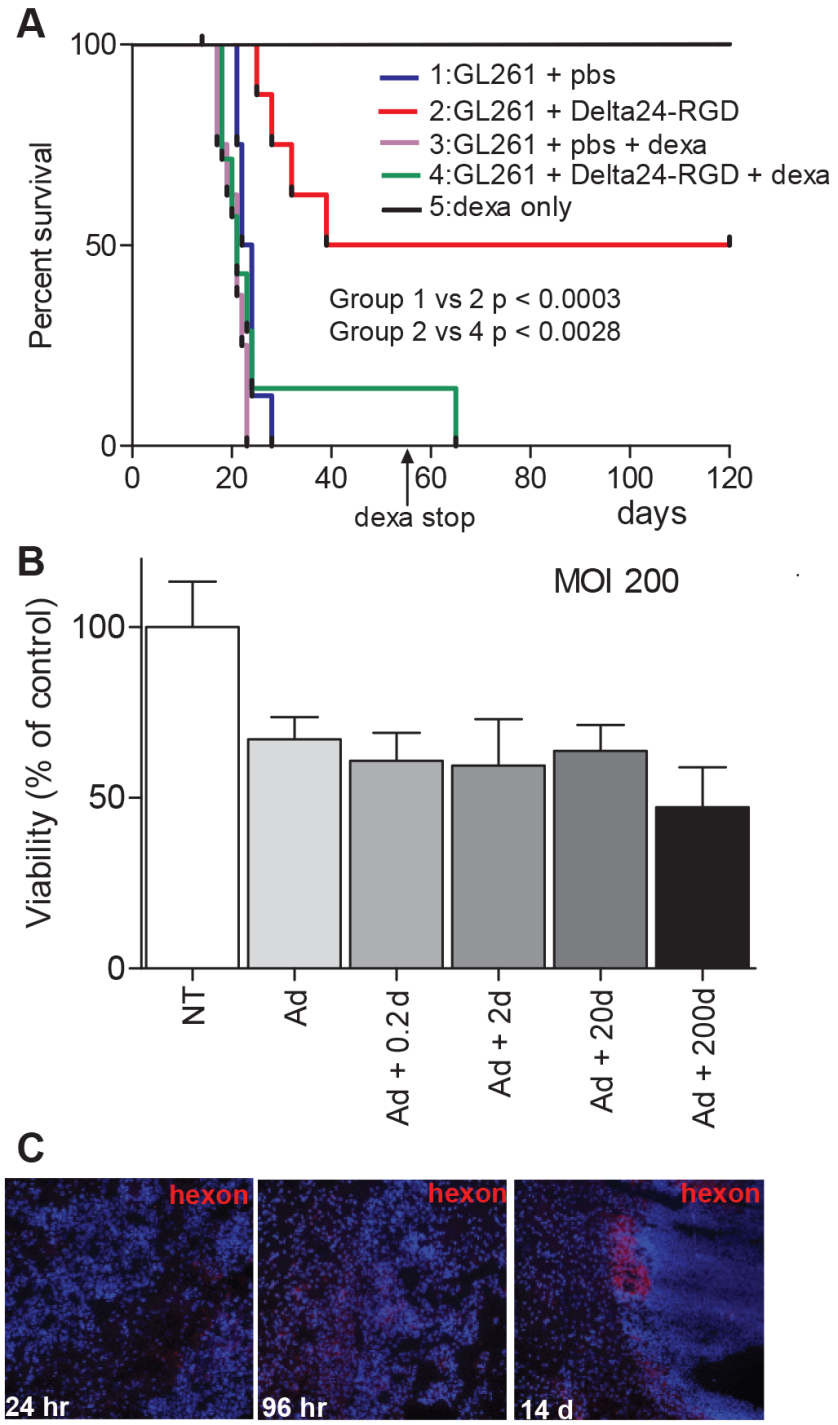
Delta24-RGD treatment results in long-term survival in the GL261 immune competent model. A) Kaplan-Meier survival plot of C57BL/6 mice injected with GL261 cells and injected five days later with Delta24-RGD (n = 8, red line), PBS (n = 8, blue line) or received dexamethasone treatment (daily 7.5 mg/kg, upon day 55 (arrow) (n = 8, light purple line) and Delta24-RGD+dexamethasone (n = 8, green line). Non-tumor bearing mice received dexamethasone as a control for dexamethasone toxicity (n = 4, black line). B) To test the effect of dexamethasone on viral efficacy in vitro GL261 cells were infected in triplicate with Delta24-RGD in the absence (light grey bar) and presence of 0.2, 2, 20 and 200 µM dexamethasone (0.2 d to 200 d). The mean viability is expressed as percentage of the control ± SD (white bar). C) In vivo staining for the adenoviral protein hexon shows a similar staining pattern with dexamethasone (10 mg/kg/day) compared to the control (shown in figure 1C). Representative images of two mice per time-point are shown.

To assess the therapeutic activity of Delta24-RGD in an immune competent model, mice bearing intracranial GL261 tumors were locally injected with 10^8^ infectious units (iu) Delta24-RGD, which induced a significant survival benefit with 50% of the treated mice (n = 8) experiencing long-term survival (figure 2A, red line compared to PBS group, blue line P<0.0003). Interestingly, the survival benefit induced by Delta24-RGD treatment was completely abolished upon daily administration of the immune suppressive agent dexamethasone (green line, p<0.0028 compared to virus alone, red line, figure 2A). Dexamethasone alone had no effect on tumor growth (purple line), and did not induce toxicity in control, non-tumor bearing mice (n = 4, black line, figure 2A).

The counteracting effect of dexamethasone on survival was not related to an inhibitory effect on virus activity in GL261 cells. The in vitro cytotoxicity of the virus was comparable in the presence or absence of (a dose range of) dexamethasone (figure 2B). *In vivo*, the pattern of tumoral hexon staining in dexamethasone co-treated mice (figure 2C) was similar to virus only treated mice (figure 1C). Together, these results suggest that the immunosuppressive agent dexamethasone does not directly affect viral activity but inhibits the immune-mediated therapeutic efficacy of Delta24-RGD.

### Delta24-RGD Induces Protective Anti-tumor Immunity

**Figure 3:**
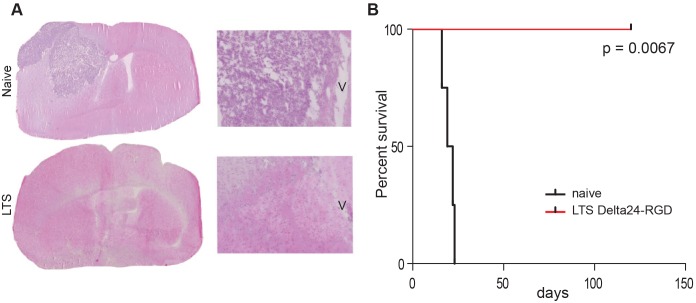
Delta24-RGD induces long term anti-tumor immunity. Long term survivors (LTS, n = 4) were rechallenged with GL261 cells. A) HE staining showing tumor growth in naïve mice and the absence of tumor in the long term survivors (LTS) (magnification 1,25x (left) and 5x (right)), V is indicating the ventricle. B) Kaplan-Meier survival plot demonstrating significant survival of rechallenged mice (log-rank test p = 0.0067).

To further investigate whether the survival benefit after Delta24-RGD treatment is immune mediated, the long-term survivors and the dexamethasone-treated control mice from the previous survival experiment were (re)challenged with GL261 cells in the contralateral hemisphere. All control mice developed tumors within 22 days while the long-term survivors from the Delta24-RGD treatment show protection against new tumor development (p = 0.0067, figure 3A, B). Analysis of the brains of these long-term surviving rechallenged mice, revealed absence of tumor cells (figure 3A). Collectively, these results indicate that Delta24-RGD treatment elicits a therapeutic and long-lasting protective anti-tumor immune response.

### Delta24-RGD Treatment is Accompanied by Local Production of Specific Cytokines and Chemokines

**Figure 4:**
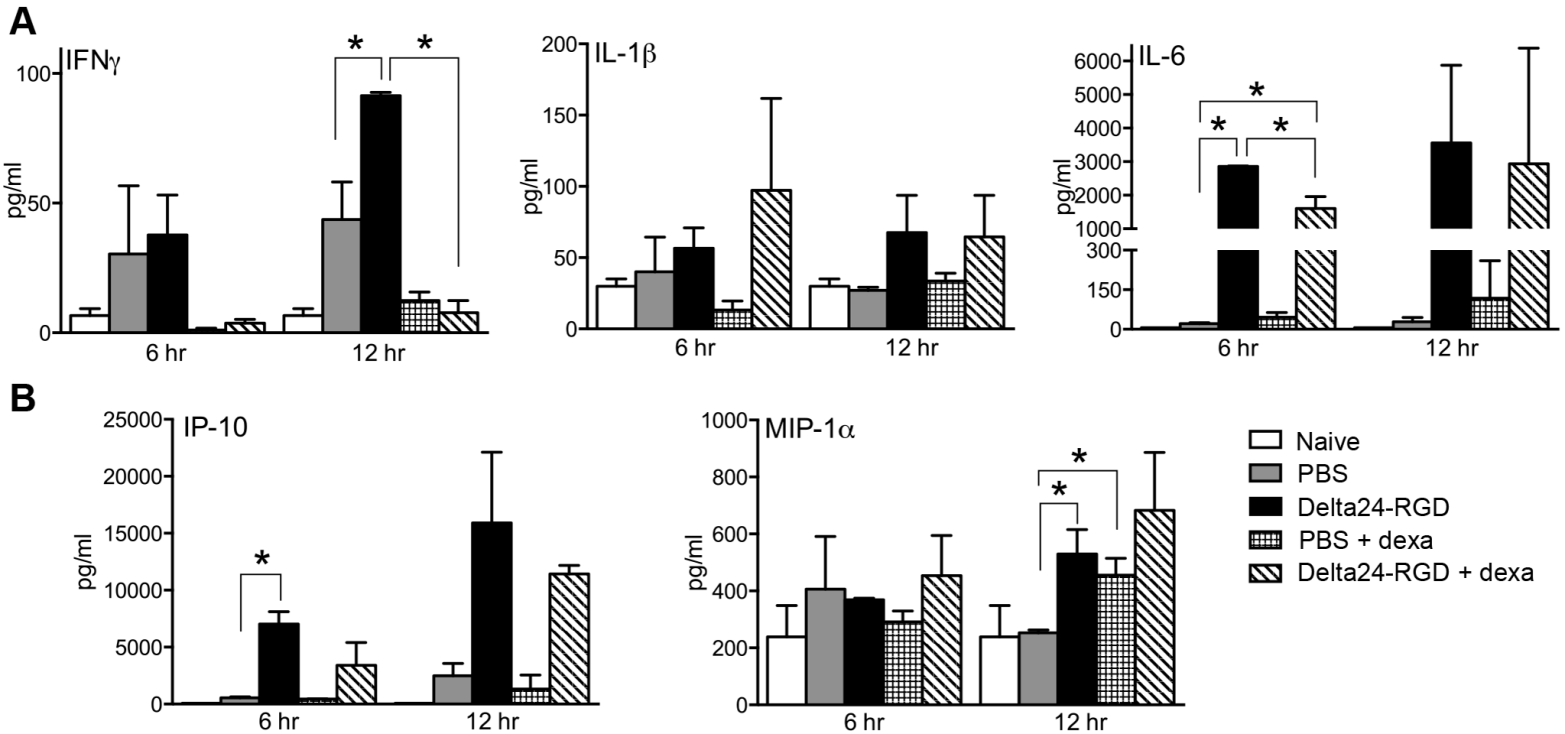
Local expression of cytokines and chemokines is upregulated after viral treatment. A) Cytokines (IFNγ, IL-1β and IL-6) and B) chemokines (IP-10 and MIP-1α) were measured in brain lysates of naïve controls (white bars), PBS-treated tumor bearing controls (grey bars), Delta24-RGD treated mice (black bars), dexamethasone co-treated controls (horizontally-striped bars) and dexamethasone-virus treated mice (diagonally-striped bars). Mean cytokine/chemokine concentrations of two brain lysates in pg/ml ± SD are shown (stars indicate p<0.05).

Following up on our findings of immune-mediated antitumor activity, we further investigated the effects of Delta24-RGD on the local production of inflammatory cytokines. In brain lysates of PBS and Delta24-RGD treated mice, we analyzed the presence of cytokines and chemokines. Levels of IFNγ, a key cytokine in viral infections, were found to be higher in tumor-bearing mice compared to non-tumor bearing mice (white bars compared to grey and black bars, figure 4A). Upon viral treatment IFNγ production was significantly upregulated compared to the control group by 12 hr (grey bar vs black bar, p<0.05). This induction was completely abrogated when mice were co-treated with dexamethasone (black bar vs striped bar, p<0.05). Of the acute phase cytokines, production of IL-1β in some of the treated mice could be detected at 12 hours (figure 4A), however this was not significant due to high variability between mice. IL-6 production increased more than 100 fold in response to viral treatment by 6 and 12 hours post treatment (grey bar vs black bar, p<0.0001). Although dexamethasone co-treatment significantly diminished this upregulation (black bar vs diagonally-striped bar, p<0.05), substantial levels of IL-6 are still present in these mice compared to the PBS-controls (grey bar vs diagonally-striped bar, p<0.05).

The chemokines IFNγ-induced protein (IP-10, CXCL10) and Macrophage Inflammatory Protein (MIP)-1α (CCL3), responsible for the recruitment of lymphocytes and monocytes, respectively, were also induced at 6 and 12 hrs post treatment (Figure 4B). This was significant for IP-10 at 6 hrs post treatment (grey vs black bar, p<0.05) and dexamethasone treatment did not affect this induction. MIP-1α levels were found to be significantly upregulated at 12 hrs in both virus-treated (p<0.05), and dexamethasone-treated (p<0.05) mice. Dexamethasone and viral treatment increased the MIP-1α levels in only a few of the mice and therefore did not reach statistical significance (p = 0.1129). Other cytokines analyzed (GM-CSF, IL-2, IL-4, IL-10, IL-12 (p70), IL-13, IL-17, VEGF and TNFα) were below detection level of the assay, or did not exhibit any significant changes upon tumor growth or Delta24-RGD treatment (results not shown).

### Delta24-RGD Infection Attracts F4/80+ Macrophages, CD4+ and CD8+ T-cells to the Tumor

**Figure 5:**
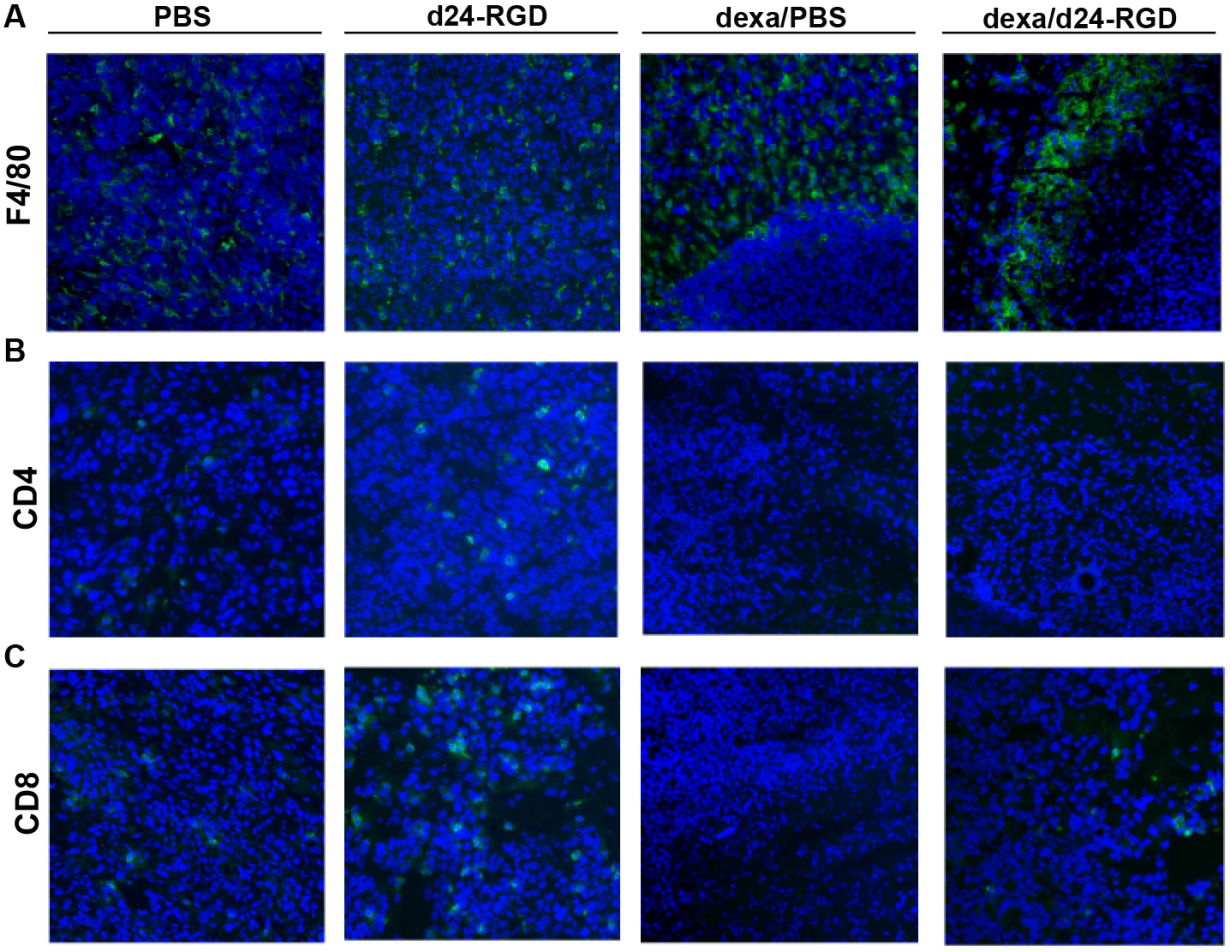
Influx and spatial distribution of F4/80+ macrophages, CD4+ and CD8+ cells. Immunohistochemical analysis of F4/80+ macrophages (A), CD4+ cells (B) and CD8+ cells (C) at 14 days post injection. Where indicated mice received 10 mg/kg/day dexamethasone (the 2 right columns). Representative images of two mice per time-point are shown (magnification 20x).

To evaluate if the induction of cytokines and chemokines upon Delta24-RGD treatment was followed by actual influx of immune cells and to visualize the spatial distribution of these cells, brains from mice were harvested for immunohistochemical analysis of F4/80+, CD4+ and CD8+ cells at 6 hours and 14 days post treatment. The F4/80+ staining for macrophages revealed that these cells are present inside the tumor at 6 hours post infection (not shown) and at 14 days both in PBS and in virus treated mice (figure 5A). Interestingly, in dexamethasone-treated mice, both with and without virus, F4/80+ cells accumulated at the periphery of the tumor with no sign of tumor infiltration.

CD4+ and CD8+ cells were not observed at 6 hours post treatment (not shown). A small increase was observed in the staining for CD4+ cells in the tumors of Delta24-RGD treated mice at 14 days, while very few CD4+ cells were detected in the dexamethasone treated mice (figure 5B). An increase in the numbers of CD8+ T cells in the Delta24-RGD-treated tumors was noted (figure 5C). Very few or no CD8+ T cells were observed in PBS-treated tumors and the numbers of CD8+ T cells were decreased in dexamethasone+virus treated tumors compared to virus alone (figure 5C).

### Treatment Efficacy in a Multifocal Model

**Figure 6:**
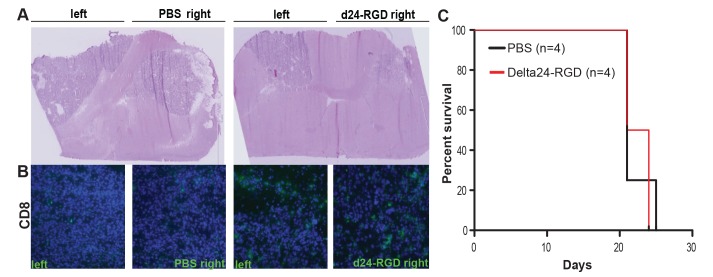
Influx of CD8+ T-cells in untreated tumor in multifocal model. A) H&E staining of multifocal tumor model showing GL261 tumors located in both hemispheres. The right-side tumor is either treated with Delta24-RGD or PBS as a control (magnification 1,25x). B) Immunohistochemical staining for CD8+ cells. Representative image of two mice per group is shown (magnification 20x). C) Survival analysis of multifocal model (not significant, Log-Rank test).

To investigate whether the immune mediated anti-tumor effects induced by Delta24-RGD treatment could also act upon tumor cells at a distance, a multifocal tumor model was set up. To this end, GL261 cells were inoculated bilaterally into both hemispheres. Five days after tumor implantation, Delta24-RGD treatment (or PBS as a control) was injected unilaterally. After 14 days mice were sacrificed to investigate the influx of CD8+ T cells. Delta24-RGD treated tumors were smaller and, as expected, contained high numbers of CD8+ cells (figure 6A, B). Interestingly, CD8+ infiltrates were also found in the contralateral tumor and to a greater extent than in PBS-treated tumors (figure 6B). Despite the influx of CD8+ T-cells in the contralateral, untreated tumor, survival of these mice was not significantly different from PBS-treated mice (figure 6C), presumably due to the large total tumor load. In this model, the untreated contralateral tumor led to symptomatic tumor burden.

### Treatment-induced CD8+ T Cells Recognize Both Virus and Tumor

**Figure 7:**
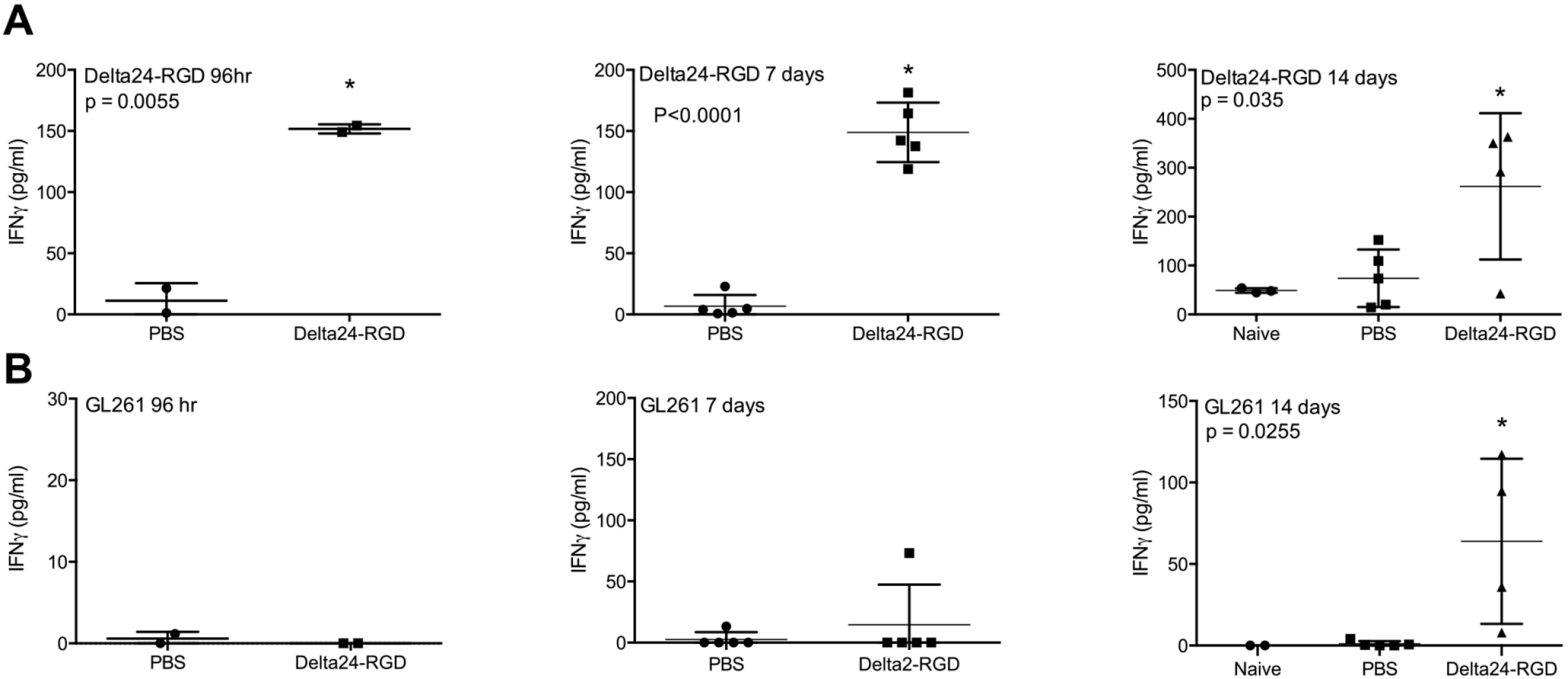
Delta24-RGD treatment of GL261 glioma induces virus and tumor specific T cells. IFNγ levels (in pg/ml±SD) in supernatants of splenocytes derived at 96 hr and 7 and 14 days after treatment from naïve, PBS and Delta24-RGD treated mice and cocultured with Delta24-RGD (A) or GL261 cells (B). The IFNγ levels were corrected for those produced by splenocytes cocultured with the control cell line A549.

To gain further insight into the Delta24-RGD-induced specific cellular immune response, splenocytes from naïve, PBS- and virus-treated mice, harvested 48 h, 96 h, 7 days and 14 days post treatment and were co-cultured with either the virus or GL261 tumor cells. IFNγ production was assessed as a marker for CD8+ T cell activation upon antigen recognition. At 48 hours post treatment, levels of IFNγ were comparable between the groups (results not shown). However, starting from 96 hours splenocytes from virus treated mice had a higher production of IFNγ after Delta24-RGD co-culture, than those from naïve or PBS-treated mice, indicating that splenocytic T cells recognize the virus (figure 7A).

In parallel, the T cell response directed to the tumor cells was assessed by co-culture of splenocytes with GL261 tumor cells. Notably, splenocytes derived from virus-treated mice at day 14 produced higher levels of IFNγ in GL261 co-culture compared to splenocytes from PBS-treated or naïve mice (figure 7B). No difference in IFNγ levels was observed between splenocytes derived at earlier timepoints (figure 7B). Co-cultures of splenocytes with lysates prepared from GL261 cells showed similar levels of IFNγ (results not shown). Together, these results indicate that local Delta24-RGD treatment elicits a specific T cell response to both the virus and the tumor. Moreover, viral specificity, detected at 96 hours, precedes tumor cell specificity, which is detected at 14 days.

## Discussion

It is relatively unexplored thusfar to what extent oncolytic adenoviruses induce an anti-tumor immune response and how this contributes to the therapeutic efficacy of the virus. Clinical trials conducted with ONYX-015, an E1B attenuated adenovirus, were mainly focused on the direct oncolytic effect in various tumors or provided anecdotal evidence of a lymphocytic infiltrate in glioma after treatment [6], [31], [32]. Recently, the anti-tumor immune response upon treatment with a GM-CSF-armed oncolytic adenovirus was reported in humans [33]. This clinical study involved various types of cancer but not glioma. With the brain traditionally considered as being an immunologically privileged organ, a concept now revisited, studies into immune-mediated therapies are of particular interest. The study presented here on oncolytic adenoviral therapy for glioma demonstrates clear evidence of a treatment-induced anti-tumor immune response that contributes to therapeutic outcome.

Using an immune competent glioma model based on the murine GL261 cells, we show that Delta24-RGD can infect, replicate and induce cytotoxicity in these glioma cells as shown in figure 1. Compared to human glioma cultures [5], about a 10-fold higher MOI is required to induce cytotoxicity in the murine GL261 glioma cells in vitro. Replication of the virus in GL261 cells is also less efficient than in human tumor cells, as has also been described for adenoviral replication in a panel of murine carcinoma cells [34]. Moreover, the life cycle of the virus seems prolonged in GL261 cells; the increase in the expression of E1A gene starts at 72 hr, while in other human glioma cell lines Delta-24RGD has a replication cycle of approximately 48 hrs [5]. Despite this reduced oncolytic potency, it is possible to mimic and study the induction and therapeutic benefit of an anti-tumor immune response in relation to oncolytic adenovirus treatment of intracranial glioma.

The GL261 model is moderately immunogenic, since only 40% of mice reject the tumor when vaccinated with GL261 cells prior to tumor injection. Vaccination post tumor injection does not influence the tumor growth in this model [30]. Delta24-RGD was administered in mice with established GL261 tumors five days after tumor cell injection. At that time point the immune suppressive environment, a known hallmark of glioma, is already established as exemplified by the presence of regulator T-cells [35], [36]. Indeed, our results show that the intratumoral influx of effector immune cells (CD8+ T-cells) is very limited in untreated circumstances (figure 5C).

In immune deficient glioma models, Delta24-RGD treatment cures up to 80% of the mice [5], [14], [37]. This effect is a direct result of the oncolytic activity of the virus. In an immune competent setting, however, interplay with the immune system is initiated. In the GL261 model, Delta24-RGD cured 50% of mice. Moreover, upon rechallenge, long-term survivors are protected against tumor formation, signifying the role of a memory immune response (figure 3). The importance of the immune system is also highlighted when it is suppressed by daily administration of dexamethasone, which completely abolishes the treatment efficacy of Delta24-RGD. In vitro, dexamethasone did not reduce Delta24-RGD-induced oncolysis (figure 2B) and staining of treated tumors for the adenoviral protein hexon showed no difference between virus-treated and dexamethasone+virus-treated mice (figure 1C and 2C). Furthermore, no cytotoxic side effects of dexamethasone were detected and tumor growth and survival in dexamethasone treated mice did not differ from PBS-treated control mice (figure 2A). Therefore, it is unlikely that dexamethasone negatively influenced viral replication or survival in this model, and it is more probable that the immunosuppressive effects of dexamethasone are responsible for hampering the therapeutic efficacy of Delta24-RGD [38], [39]. This is also consistent with the reduced intratumoral influx of CD4+ and CD8+ T-cells (figure 5B, C) and the diminished production of IFNγ (figure 4A) in the brains of dexamethasone treated mice. These results are in line with other reports showing diminished lymphocyte infiltration in the brain tumor area during dexamethasone treatment [40], [41].

Interestingly, with dexamethasone treatment F4/80+ macrophages are also no longer present in the central tumoral area but are located at the border of the tumor. This phenomenon has also been described with cyclophosphamide administration and HSV oncolytic virotherapy in a rat glioma model, where the peripheral macrophages were restrained at the border of the tumor whereas brain resident macrophages/microglial cells accumulated in the core of the tumor [21], [42]. The reported effects of dexamethasone on tumor vascular permeability may play a role in hampering the influx of peripheral macrophages [43]. Taken together, these results may have implications for clinical trials testing (adenoviral) OVs. Dexamethasone is commonly prescribed during glioma management, albeit at lower dosages than used in the current mouse study, and may negatively affect development of immune anti-tumor activity. Also, the concomitant use of chemotherapy with Delta24-RGD may warrant caution as the generalized induction of cytopenia and immune cell depletion may hamper the anti-tumor response elicited by Delta24-RGD treatment [44].

Treatment with Delta24-RGD creates a pro-inflammatory environment in the tumor by the upregulation of several cytokines, chemokines and the recruitment of F4/80+ macrophages, CD8+ and CD4+ T cells (figure 4, 5). The cytokines induced upon viral treatment, have previously been described upon systemic or direct injection of adenoviral vectors in the brain and are presumed to be a direct effect of the immune response towards adenoviral capsid proteins [45], [46]. This response is regulated via the complement system [47] and is Toll-like receptor (TRL) 2 and TRL9 dependent [48]. We hypothesize that, together, these danger signals induced by the virus are able to tip the balance from the immune suppressive environment induced by the glioma [36], towards a more pro-inflammatory condition in which immune cells are attracted to the tumor and an anti-tumor response can be elicited. This condition is not only induced in the virus-injected tumor, but it also able to extend to tumor cells at a distance, as is shown in our multifocal model (figure 6). The highly infiltrative nature of human gliomas, with individual tumor cells at large distance from the tumor core, makes this an important feature of adenoviral OV therapy. Indeed, in the multifocal model, Delta24-RGD treatment of the tumor in the right hemisphere led to the infiltration of CD8+ T cells in the contralateral tumor. Despite these effects, we did not observe a survival benefit of Delta24-RGD treatment. This is most likely due to the aggressive nature of the GL261 model (mean survival 20 days) and the fact that the development of a T-cell mediated anti-tumor response takes between 7 and 14 days (figure 7). During this time period, the untreated tumor, not affected by direct viral oncolysis, continues to grow rapidly and animals succumb to the tumor burden.

The intratumoral influx of CD8+ cells together with the locally increased production of IFNγ, the functional T-cell response against the tumor, and the protective immunity against tumor rechallenge, point towards an essential role of CD8+ T-cells during this treatment. These results urge further research into characterization of this specific response and the development of strategies to further enhance it. This will greatly increase therapeutic options, e.g. by arming OVs with critical chemo- or cytokines, combining OV treatment with either TCR-modified T-cells [49], [50] or with antibodies targeting T-cell costimulatory molecules [51].

## Methods

### Cell Culture

The mouse glioma cell line GL261, obtained from NCI Tumor Repository (Frederik, MD), the human lung carcinoma cell line A549 (ATCC, Manassa, VA) and the 911 cell line [52] (kindly provided by dr. RC Hoeben, Leiden University, The Netherlands) were all maintained in Dulbecco’s Modified Eagle Medium (Invitrogen, Carlsbad, CA) supplemented with 10% fetal calf serum (FCS; Invitrogen) and 1% penicillin/streptomycin (Invitrogen).

### Viruses

The human Delta24-RGD virus was previously described by Suzuki et al 2001 [17]. The Ad-luc-RGD virus was kindly provided by dr DT Curiel, (University of Alabama Birmingham, Alabama). Viral stocks were produced as described previously [53]. When cytopathic effect appeared, cells and supernatants were pooled and the virus was purified using the AdEasy Virus Purification Kit (Stratagene, La Jolla, CA). The virus titer was determined on A549 cells for Delta24-RGD or 911 cells for Ad-luc-RGD using the Adeno-X Rapid Titer Kit (Clontech, Mountain View, CA).

### *In vitro* Cytotoxicity and Viral Load Assay

For all experiments, GL261 cells were seeded in a flat-bottom 96-well plate (Corning, NY) at a density of 5×10^3^/well. For the cytotoxicity test, cells were infected 24 hr after plating with Delta24-RGD MOIs ranging from 200 to 800 in triplicate. Cell viability was assessed at day 3, 6 and 8 using the WST-1 reagent (Roche, Basel, Switzerland) according to the manufacturer’s instructions. To assess the effects of dexamethasone on Delta24-RGD oncolytic activity, 0.2, 2, 20 and 200 µM dexamethasone (hospital pharmacy, ErasmusMC, Rotterdam, The Netherlands) was added 24 hr prior to and simultaneously with Delta24-RGD infection with MOI 200. To assess if Delta24-RGD was able to replicate in GL261 cells, the viral load was determined by quantitative PCR of the adenoviral E1A gene. Therefore, 5000 GL261 cells were infected with 100 MOI and 24, 48, 72 and 96 hours after infection, cells and supernatants were harvested separately. The cells were lysed by 3 freeze/thaw cycles. Nucleic acids were extracted both from the lysed cells and supernatant using the High Pure Viral Nucleic Acid extraction kit of the Magnapure LC (Roche Molecular Systems, Switzerland). Amplification was performed using the 2x Taqman Universal Mastermix (Life Technologies) and primers add24-RGDfwd (5′-acactaaacggtacacaggaaacag-3′) and add24-RGDrev (5′-gccagaccagtcccatgaaa-3′) and FAM-BHQ labelled probe add24-RGDprobe (5′-ccgcggagactgtttctgccca-3′). Real time PCR amplification was read on a Lightcycler 480 (Roche Molecular Systems). In parallel, a calibration curve of a Delta24-RGD stock with a known viral particle titre was run.

### Intracranial Immune Competent Mouse Model

All animal experiments described in this paper have been conducted according to Dutch guidelines for animal experimentation. All animal experiments were reviewed by and performed with approval of the Erasmus MC Animal Ethics Committee (DEC) of the Erasmus Medical Center, Rotterdam, The Netherlands (Protocols DEC EMC-1688, DEC EMC-2100 and DEC EMC-2707). The mice were housed in individually ventilated cages with sterile bedding, water, and rodent chow. All efforts were made to minimize animal suffering. No more than mild or moderate discomfort of animals was expected from the treatments, and no unexpected discomfort was observed.

Female C57BL/6 mice (6–9 weeks old, Harlan, Horst, The Netherlands) were stereotactically injected with 5×10^4^ GL261 cells 3 mm deep in the right hemisphere (2.2 mm lateral, 0.5 mm posterior of bregma) as described previously [27]. In the rechallenge experiment, 5×10^4^ GL261 cells were injected stereotactically in the left hemisphere. For the multifocal tumor model, 5×10^3^ GL261 cells were injected in the left and right hemisphere at the same coordinates. Dexamethasone treatment (10 mg/kg/day or 7.5 mg/kg/day i.p. as indicated in the figure legends) was started one day before intracranial injection with GL261 cells. In the survival experiment animals received dexamethasone for 55 days. Five days after GL261 injection, the same burr hole was used to inject 10^8^ pfu Delta24-RGD or 1,25% glycerol (Sigma)/PBS as control. All intracranial injections (tumor cells or virus) were performed under isofluorane inhalational anesthesia and during and directly after the intracranial injections mice received additional local analgesia (0.25% Bupivacaine) on the head wound. For the early analysis timepoints mice were euthanized at 6, 24, 48, 96 hr, 7 or 14 days post treatment by cervical dislocation under isofluorane anesthesia. For the survival experiments mice were monitored daily and were euthanized upon more than 20% weight loss or when neurological symptoms appeared. Brain, spleen and blood were harvested for further studies.

### Neutralizing Antibodies

Determination of neutralizing antibodies was done as described by Sprangers et al [54]. Briefly, serum was collected from virus treated mice at 48, 96 hr and 7 and 14 days post-treatment. A549 cells were plated at 10^4 ^cells in 96-well flat-bottom plates and infected with the Ad-luc-RGD virus (MOI 100) in the presence of a serial dilution of the sera taken at the various timepoints. After 24 hours of incubation the cells were lysed using 0.9% Triton-X100 (Sigma-Aldrich) and total luciferase was measured using the Luciferase Assay System (Promega) according to the manufacturer instructions. Results are presented in RLU as percentage of inhibition compared to control infection (A549 cells infected with Ad-luc-RGD) levels.

### Cytokines

Proteins were isolated from snap-frozen mouse brains as described by Datta et al [55]. In brief, the right tumor-containing hemisphere was disrupted and the Bioplex Cell Lysis Buffer (Bio-rad, Hercules, CA) was added. The homogenates were agitated on ice for 30–40 minutes to allow complete lysis. The supernatants were collected and protein concentration was determined using the BCA Protein Assay Reagent Kit (Roche). Selected cytokines and chemokines were measured using the Milliplex Map Mouse Cytokine/Chemokine Magnetic Bead Panel (Millipore). Analysis was done with the Milliplex Analyzer 3.1 xPonent System (Millipore).

### Immunohistochemistry

Cryosections of snap frozen brains were made and fixed with ice-cold acetone. Proteins were blocked for 10 min with Protein Block (Dako, Glostrup, Denmark) and stained with the following primary antibodies: rat anti-mouse F4/80 (Bio-Connect, Huissen, The Netherlands), rat anti-mouse CD4 (Biolegend, San Diego, CA), rat anti-mouse CD8 (eBioscience, San Diego, CA) and goat anti-adenovirus (Millipore, Billerica, MA). Secondary antibodies ALEXA Fluor-546 rabbit anti-goat IgG (H+L) (Invitrogen) and ALEXA Fluor-488 goat anti-rat IgG (H+L) (Invitrogen) were used to detect the primary antibodies. All sections were counterstained with 4′,6 Diamidino – 2 – Phenylindole dihydrocholoride (DAPI) and mounted (Vectashield, Vector Laboratories, Burlingame, CA). Images were merged using ImageJ software (Rasband, W.S., ImageJ, U.S. National Institute of Health, Bethesda, Maryland, USA, http://imagej.nih.gov/ij/, 1997–2012).

### Detection of Reactive Splenocytic T Cells

T cell reactivity in spleens was monitored as described by Pouw and colleagues [56]. In short, spleens were mechanically dissociated and erythrocytes were removed using a NH_4_CL solution. Splenocytes were maintained in complete mouse medium containing RPMI 1640 w/25 mM Hepes and L-Glutamine (Invitrogen) supplemented with 10% FCS, 1% penicilin/streptomycin, NEAA (Lonza, Basel, Switserland), 1 mM NaPyr (Invitrogen) and 50 µM β-mercaptoethanol (Sigma-Aldrich, St. Louis, MO). The splenocytes were stimulated with Concanavalin A (2.5 µg/ml, Sigma-Aldrich) and 100 U/ml human recombinant IL-2 (Proleukin, Chiron, Amsterdam, The Netherlands) for 48 hours. To assess IFNγ production, 10^6^ stimulated splenocytes were co-cultured with 10^4^ GL261 cells, A549 cells, and Delta24-RGD. Supernatants were harvested and the level IFNγ was determined with OptEIA ELISA kit II (BD Biosciences). Levels of IFNγ produced in the co-culture of splenocytes and GL261 cells were corrected for those produced in the co-culture of splenocytes with A549 cells and considered as background levels.

### Statistical Analysis

Statistical analysis was done using the Prism Graphpad Software (Graphpad Software Inc. La Jolla, CA). For the survival experiments the log-rank test was used, for the other experiments the student’s t-test. Differences were considered statistically significant when p<0.05.
